# The Waterlogging Tolerance of Wheat Varieties in Western of Turkey

**DOI:** 10.1100/2012/529128

**Published:** 2012-04-29

**Authors:** Ilkay Yavas, Aydin Unay, Mehmet Aydin

**Affiliations:** Kocarli Vocational High School, Adnan Menderes University, 09100 Aydın, Turkey

## Abstract

This research was conducted to determine the wheat varieties against waterlogging which was clearly increased in recent years. For this purpose, this study was performed at Field Crops and Soil Science Department of Agricultural Faculty of Adnan Menderes University during wheat growth stages of 2007-2008. The experimental design was randomized complete block design with split split plot arrangements. The main plots were temperature applications (heat and normal), the growth periods (Zadoks scale; GS14, GS32, GS14 + GS32, and control) were split plots and varieties were split-split plots. The eight different wheat varieties were evaluated in the pots. The waterlogging was performed during GS14, GS32 and GS14 + GS32. In a pot experiment, plants were subjected to waterlogging to the soil surface for 10 days. All applications and varieties decreased the single plant yield. The waterlogging caused a yield loss compared with wheat grown on well-drained soil. In this study, the crop loss due to waterlogging is highly temperature dependent. The severity of the effects of the waterlogging depends on the growth stage of the plot. When all applications were compared with control by means of yield performance, Sagittario and Basribey varieties were less affected than the others.

## 1. Introduction

Cereals are the basic products used in human nutrition. Wheat is great importance in cereals. The world annual wheat production is around 689 million tons and plant area is 223 million hectares. The wheat production is 17.8 million tons and plant area is 7.6 million hectares in Turkey [1].

Worldwide, about 10% of all irrigated land suffers from waterlogging. Soaking of agricultural land is caused by a rising water table or excessive irrigation. Waterlogging compacts soil, deprives roots of oxygen, and contributes to salinization. Waterlogging is common in wheat following rice, with plants turning yellow due to oxygen stress. Waterlogging has been shown to limit wheat yields in many regions of the world; an area about 10 million ha is waterlogged each year in developing countries [2].

Wheat has high adaptability for all kinds of climates and regions. Waterlogging which causes oxygen deficieny, will prevent to root and shoot growth, reduce the accumulation of dry matter and as a result of these yield will reduce. Especially, February and March rainfalls in western Turkey lead to the breakdown of cultivated fields and reduction of the yields. Wheat (*Triticum aestivum* L.) is one of the most intolerant crops to soil waterlogging [3]. Prolonged periods of rainfall combined with poor soil drainage often cause low oxygen in the soil. Decrease in soil O_2_ content, under flooded conditions, is often accompanied by increase in soil CO_2_ and ethylene content. Such changes have detrimental effects on root and shoot growth of wheat [4]. Productivity from soils susceptible to waterlogging may be increased by drainage and the introduction of waterlogging-tolerant genotypes [3]. High temperatures tend to exacerbate the negative effects of waterlogging. Yield is affected differently, depending on the crop's stage of development at the time stress is applied [5]. On waterlogged sites, plants show chlorosis and necrotic spots on older leaves. Both Mn toxicity and N deficiency may be induced by the low redox potential in waterlogged soils that produces plant-available Mn^2+^ and promotes denitrification of NO_3_^−^. Under these anaerobic conditions, root metabolism and root growth are inhibited, since the lack of O_2_ affects the energy status of the plant [6].

The colder soil temperatures associated with waterlogging in winter-wheat growing areas reduce the amount of oxygen required for root respiration. Thus yield reductions associated with waterlogging in colder areas are not as great as those in the more temperate and tropical areas of the world [7]. Similarly, some studies show that soil oxygen decline under waterlogging is rapid at most temperature ranges [8]. Chlorosis of lower leaves [9], decreased plant height and lower number of spike-bearing tillers [10], reduced root and shoot growth [11] in the wheat waterlogging areas. The uptake of trace metals by two plant species (French bean and maize) has been measured on two soils subjected to various waterlogging regimes. Uptake of both manganese and iron was increased due to soil waterlogging, although reoxidation of the soil affected iron more than manganese. The abilities of these species to take up trace metals from soil followed the pattern predicted by selective extraction of soil for manganese, iron, and cobalt, but not for zinc and copper [12]. In waterlogged acidic soil, shoot concentrations of aluminum (Al), manganese (Mn), and iron (Fe) increased by two- to 10-fold, and in some varieties they were above critical concentrations compared with plants in drained soil. These elements decreased or remained the same in shoots of plants grown in waterlogged neutral soil [13]. The greater P uptake per unit of root biomass was a consequence of (1) an increase in soil P availability induced by waterlogging, (2) a change in root morphology, and/or (3) an increase in the intrinsic uptake capacity of each unit of root biomass. Soil P content was higher during waterlogging periods and the roots of waterlogged plants showed a higher physiological capacity to absorb P. Soil P availability was higher during waterlogging periods, roots of waterlogged plants showed a morphology more favorable to nutrient uptake (finer roots) and these roots showed a higher physiological capacity to absorb P [14].

Waterlogging is a serious environmental stress on winter wheat grown in western Turkey. In this study, the aim was the determination of tolerant varieties to waterlogging conditions, effects of temperature, and growing stages.

## 2. Materials and Methods

This study was conducted in 2007-2008 at the Field Crops Department of the Faculty of Agriculture at Adnan Menderes University in Aydin, Turkey. Aydin province is situated at 37° 39′ E and 27° 52′ N in the west Aegeon Region of Turkey, and typical Mediterranean climatic conditions are dominant in Aydin. To evaluate losses from waterlogging under pot experiments, 8 wheat genotypes were grown under no waterlogging (control) and 10 days of continuous flooding, GS14, GS32, and GS14 + GS32 [15]. After flooding the pots were allowed to come normal dry conditions by evaporation and plant use but without draining. Sufficient water was applied to waterlog from the top down, by applying water in excess of the rate at which it could infiltrate the soil, indicated by surface pooling [16]. Soil iron and soil manganese content (DTPA analysis, [17]), and soil phosphorus content [18] were determined.

The experimental design was randomized complete block design with split-split plots. The main plots were temperature applications, the growth plots were Split Plots and varieties were split-split plots. Data on plant height, tiller number, root biomass, shoot biomass, single plant yield, soil iron content, soil manganese content, and soil phosphorus content were taken on five plants per pot, and means were used for data analysis. The experiments were designed in split-split plot design with 3 replications. To increase the temperature, low tunnels were used. The growing conditions (low tunnel and normal) were the main plots, growing periods (GS14, GS32, and GS14 + GS32) were the subplots and cultivars (Golia, Gonen, Basribey, Adana 99, Cumhuriyet 75, Sagittario, Pamukova, Negev) were sub-subplots. The wheat varieties were sown on 27.11.2007 and harvested at 23.05.2008. At maturity, plants were harvested and threshed manually. The data regarding plant height, tiller number, root biomass, shoot biomass, single plant yield, soil iron content, soil manganese content and soil phosphorus content were recorded. The significance of main effects and interactions was determined at the 0.05 and 0.01 probability levels by the *F*-test. Means of the significant (*P* ≤ 0.05) main effects and interactions were separated using Fischer's protected LSD test at *P* = 0.05. The data were statistically analyzed by using a standard analysis of variance technique for a split-split plot design using Tarist software [19].

## 3. Results

The soil iron, manganese, and phosphorus contents were given at Table 1. The difference between heat and normal conditions for soil Fe and the accumulation of Fe and Mn for different growing stages were significant (Table 1).

The soil Fe content in normal condition was found significantly higher compared with heat condition (Table 2). During waterlogging, O_2_ in the soil is rapidly depleted, and the soil may become hypoxic or anoxic within a few hours. Moreover, some waterlogged soils become rich in Mn^2+^ and Fe^2+^. Waterlogging stress leads to changes in soil conditions which may affect plant growth through oxygen deficiency, reduced nitrogen availability, or manganese toxicity and so on [20].

Table 3 showed the results of variance analysis of the plant height, tiller number, shoot biomass, root biomass, and single plant yield. The results showed that the stage and cultivar factors and their interactions were found significant except for root biomass at growing stage factor.

Table 4 was that the the results of variance analysis for heat conditions. The results showed that stage and cultivar interactions were significant except for root biomass. And the root biomass for different stage was nonsignificant. Also, the differences among wheat varieties were significant for observed characters in high temperature.

The waterlogging conditions reduced all cultivar's height in GS14 + GS32 than control conditions. The tiller number at different stage was given at Table 5. The waterlogging reduced the number of tiller in wheat. For all treatments, grain losses were much less than expected from the extent of tiller loss in winter, losses after single waterlogging events ranged from 2% (after 47 days with the water table at 5 cm) to 16% (after 80 days with the water table at the soil surface). Yield losses after three waterlogging at the seedling, tillering, and stem elongation stages of growth were additive, and totaled 19% [21]. Transient waterlogging during winter and spring reduces wheat yield. Yield reductions from waterlogging are associated with reduced production and survival of tillers, fewer and smaller fertile tillers, and smaller grain size [22]. Waterlogging induced a transient N deficiency. The N concentration of the youngest expanded leaf on the mainstem and tillers declined markedly during waterlogging, but its recovery 14 days after the waterlogging was ended was independent of treatment, reaching the critical minimum concentration of 3.5%. The growth of primary tillers 1 and 2 was severely inhibited by waterlogging while the exertion of new tillers was delayed by 9 days [23].

**Figure 1:**
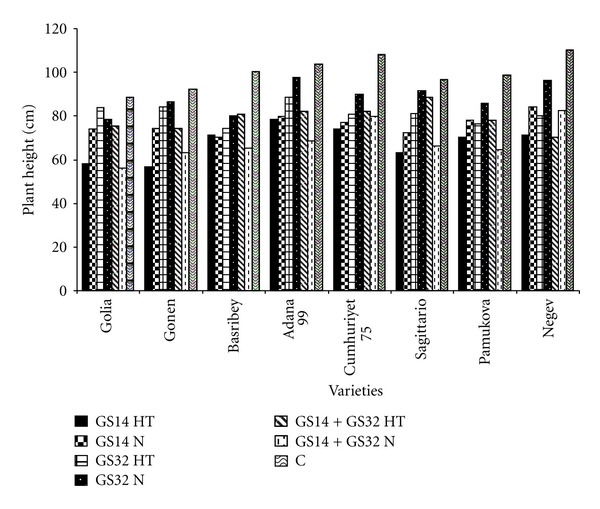
The plant height at GS14, GS32, GS14 + GS32, and control. HT: high temperature, N: normal, C: control.

Figure 1 pointed out plant height at different stages of wheat. Waterlogging significantly reduced plant height and tillering, delayed ear emergence, and resulted in 8, 17, 27, and 39% reduction in grain yield, respectively [24].

**Figure 2:**
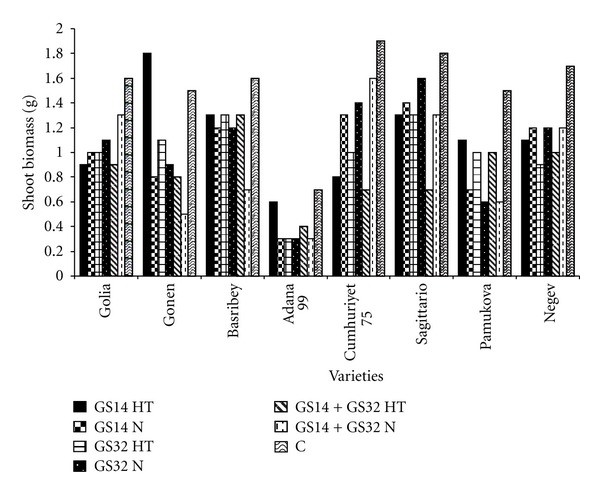
Shoot biomass at GS14, GS32, GS14 + GS32, and control. HT: high temperature, N: normal, C: control.

Figure 2 showed shoot biomass at different stage of wheat. The waterlogging conditions reduced the shoot biomass values.

**Figure 3:**
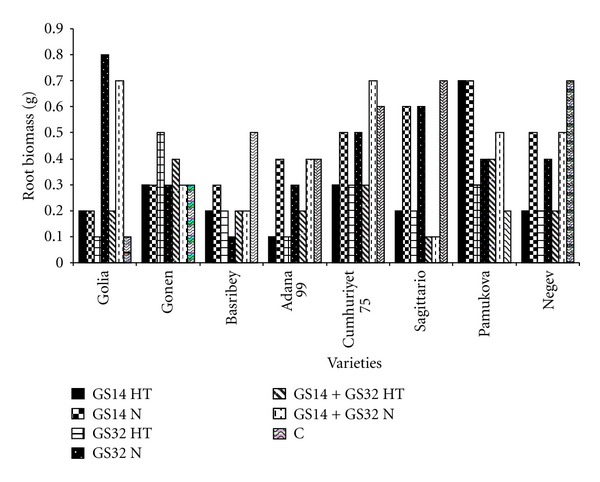
Root biomass at GS14, GS32, GS14 + GS32, and control. HT: high temperature, N: normal, C: control.

The other important characteristic for waterlogging is root biomass examined at Figure 3. The lowest root biomass value was found in Adana 99 at GS14. On the other hand the best cultivar was Cumhuriyet 75. Doubled haploid lines grown in waterlogged soil for 49 days ranged from 82% reductions in biomass at the end of waterlogging in acidic soil [25].

**Figure 4:**
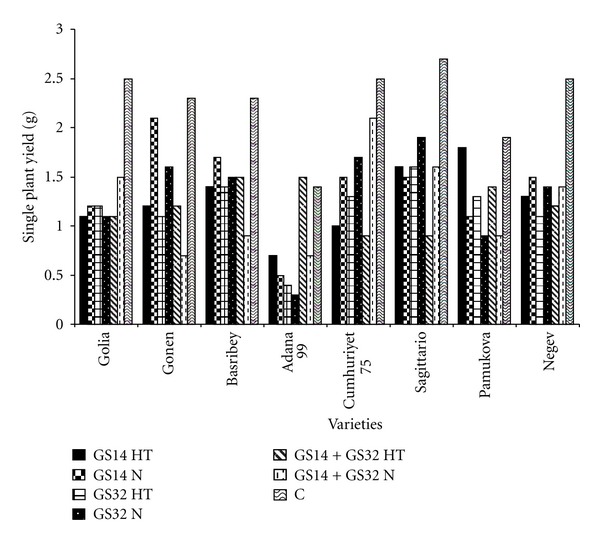
Single plant yield at GS14, GS32, GS14 + GS32, and control. HT: high temperature, N: normal, C: control.

The single plant yield values for different stages were given at Figure 4. Results indicated that significant linear responses were found for yield. Reduction of the number of spike after the waterlogging caused a significant decrease of single plant yield.

## 4. Discussion

Sparrow and Uren [26] and Wagatsuma et al. [27] stated that soil Mn concentration that could be toxic to plant growth increased in waterlogging. Similarly, mineral Fe coating of epidermal surface of roots increased under waterlogging [28]. In addition, Belford et al. [29] revealed that decreased soil oxygen was generally greater at warmer temperature. Also, in our study the soil Fe and Mn contents of nonwaterlogging pots (control) had significantly lower values, whereas waterlogging applications in both jointing and tillering significantly increased soil Fe and Mn contents. The differences in soil Fe and Mn between conditions and growing stages prepared a suitable condition to evaluate wheat varieties response to waterlogging.

There are significant differences in waterlogging tolerance for different growing stages. Diversity occurs in the timing, duration, and severity at waterlogging stress [30].

The highest plant height was found in Adana 99, Golia, Gonen, Sagittario, Basribey and Pamukova were negatively affected in the GS14 (high temperature) and GS14 + GS32 (normal conditions) for plant height. The effects of waterlogging on the Basribey and Adana 99 occurred in the GS14 + GS32 (normal conditions) negatively affected in the early growing stages and increased temperatures (GS14; high temperature). Negev was influenced by waterlogging which occurred during GS14 and GS32 periods, together with heat application. Flooding and increased temperature application were most significant effects for all cultivars. Losses caused by waterlogging need to be measured to determine the importance of traits used as waterlogging tolerance indicators. Kernels per spike and plant height had a less severe reduction from waterlogging, about 30 and 19%, respectively [31].

Taeb et al. [32] revealed that tiller number were decreased during waterlogging, tiller production, shoot dry matter, and root penetration were used for screening Tritieae species for tolerance. When these criteria were used, many wild species expressed a level of tolerance to waterlogging that was better than that of wheat. 

By the end of the experiment, shoot mass remained lower in plants from waterlogged treatments compared with continuously drained control, due to lower tiller numbers. Comparisons between alternately waterlogged and continuously waterlogged plants showed that in the alternaely waterlogged plants, shoot weights were heavier [3].

The lowest yield was observed in Adana 99 which was exposed to waterlogging at GS32. Correspondingly, the highest value was obtained from Cumhuriyet 75 at GS14 + GS32. In a field study, wheat (*Triticum aestivum* L.) crop to waterlogging for 1, 2, 4, and 6 days at the time of first irrigation (25-day-old plants) significantly reduced tillering and plant height, delayed ear emergence, and resulted in 8, 17, 27, and 39% reduction in grain yield, respectively [24].

The wheat varieties used as material showed different response to waterlogging. The significant negative effects of waterlogging on cultivars were both tillering and jointing stage (GS14 + GS32) with high temperature. Soil Fe and Mn contents significantly increased in tillering and jointing stage waterlogging applications. Basribey and Sagittario wheat varieties should be suggested as waterlogging tolerance varieties. This study will play an important role for wheat growers in the future as they will be able to make better crop management decisions when they encounter incidence of waterlogging.

Huang et al. [11] observed that waterlogging reduced shoot nitrogen content, shoot, and root growth.

## Figures and Tables

**Table 1:** The variance analysis results of soil Fe, Mn, and P contents at three growing stages in different waterlogging applications.

		Mean squares		
Source	df	Fe	Mn	P
Conditions	1	144.95*	0.484	32.711
Growing stage	3	89.78*	58.41**	267.18
Interaction	3	35.89	4.54	30.58
Error	64	32.07	11.28	103.83

Total	71			

*P < 0.05, **P < 0.01.

**Table 2:** The average values of soil Fe, Mn, and P contents at three growing stages in different waterlogging applications.

		Fe	Mn	P
Conditions	Heat	19.85 b*	11.70	27.46
	Normal	22.69 a	11.15	26.12
	LSD_(0.05)_	2.67		

Growing	Tillering + Jointing (GS14 + GS32)	23.52 a	13.45 a	28.83
Stages	Jointing (GS32)	22.10 ab	11.21 ab	24.49
	Tillering (GS14)	20.77 ab	11.91 ab	31.10
	Control	18.68 b	9.11 c	22.73
	LSD_(0.05)_	3.78	2.33	

*Within each column, means followed by a different letter are significantly different at 5% level.

**Table 3:** Results of variance analysis for some characters in normal conditions.

		Means of square				
	df	PH	TN	SB	RB	SPY
Stage	3	4645.7**	15.100**	1.834**	0.001	5.567**
Cultivar	7	423.802**	2.645**	1.789**	0.146**	1.949**
Stage × Cultivar	21	51.102**	1.063**	0.075**	0.127**	0.262**

*P < 0.05, **P < 0.01. Plant height, TN: tiller number, SB: shoot biomass, RB: root biomass, SPY: single plant yield.

**Table 4:** Results of variance analysis for heat conditions.

		Means of square			
	df	PH	SB	RB	SPY
Stage	3	4207.480**	2.082**	0.005	6.556**
Cultivar	7	199.154**	0.767**	0.093*	0.561**
Stage × Cultivar	21	102.052**	0.112**	0.033	0.293**

*P < 0.05, **P < 0.01. Plant height, SB: Shoot Biomass, RB: Root Biomass, SPY: Single plant yield.

**Table 5:** The tiller number at GS14 and control.

	Growing stage 14		
	Heat temperature	Normal	Control
Golia	0	0	3
Gonen	3	0	2
Basribey	0	0	3
Adana 99	0	0	2
Cumhuriyet 75	2	0	4
Sagittario	2	0	6
Pamukova	0	0	4
Negev	3	1	6

## Abbreviations

GS14: growing stage 14
GS32: growing stage 32
GS14 + GS32: growing stage 14 + 32.
